# Effectiveness of inhaled furosemide for acute asthma exacerbation: a meta-analysis

**DOI:** 10.1186/s13054-014-0621-y

**Published:** 2014-11-24

**Authors:** Ryota Inokuchi, Ai Aoki, Yuta Aoki, Naoki Yahagi

**Affiliations:** Department of Emergency and Critical Care Medicine, The University of Tokyo Hospital, 7-3-1 Hongo, Bunkyo-ku, Tokyo 113-8655 Japan; Department of Emergency Medicine, JR General Hospital, Yoyogi, Shibuya-ku, Tokyo 151-8528 Japan; Department of Psychiatry, Tokyo Metropolitan Health and Medical Treatment Corporation, Ebara Hospital, Ota-ku, Tokyo 145-0065 Japan; Department of Neuropsychiatry, Graduate School of Medicine, The University of Tokyo, Bunkyo-ku, Tokyo 113-8655 Japan

As the effectiveness of beta-agonists for treating asthma attacks has been established, numerous other supportive treatments for asthma attacks have also been investigated, such as systemic glucocorticoids and magnesium. Among these additional therapies, inhaled furosemide is of particular interest; several studies have evaluated the effects of prophylactic inhaled furosemide in attenuating bronchoconstriction and asthma attacks. To determine the efficacy of inhaled furosemide during asthma attacks, we performed a systematic review using the MEDLINE, EMBASE, Web of Science, and Cochrane Library databases from their inception through 14 March 2014. A meta-analysis was conducted by calculating the standardized mean difference from each study and integrating these means using a random effects model. In addition, subanalyses were performed in the studies that evaluated the peak expiratory flow rate and the forced expiratory volume in 1 second.

**Figure 1:**
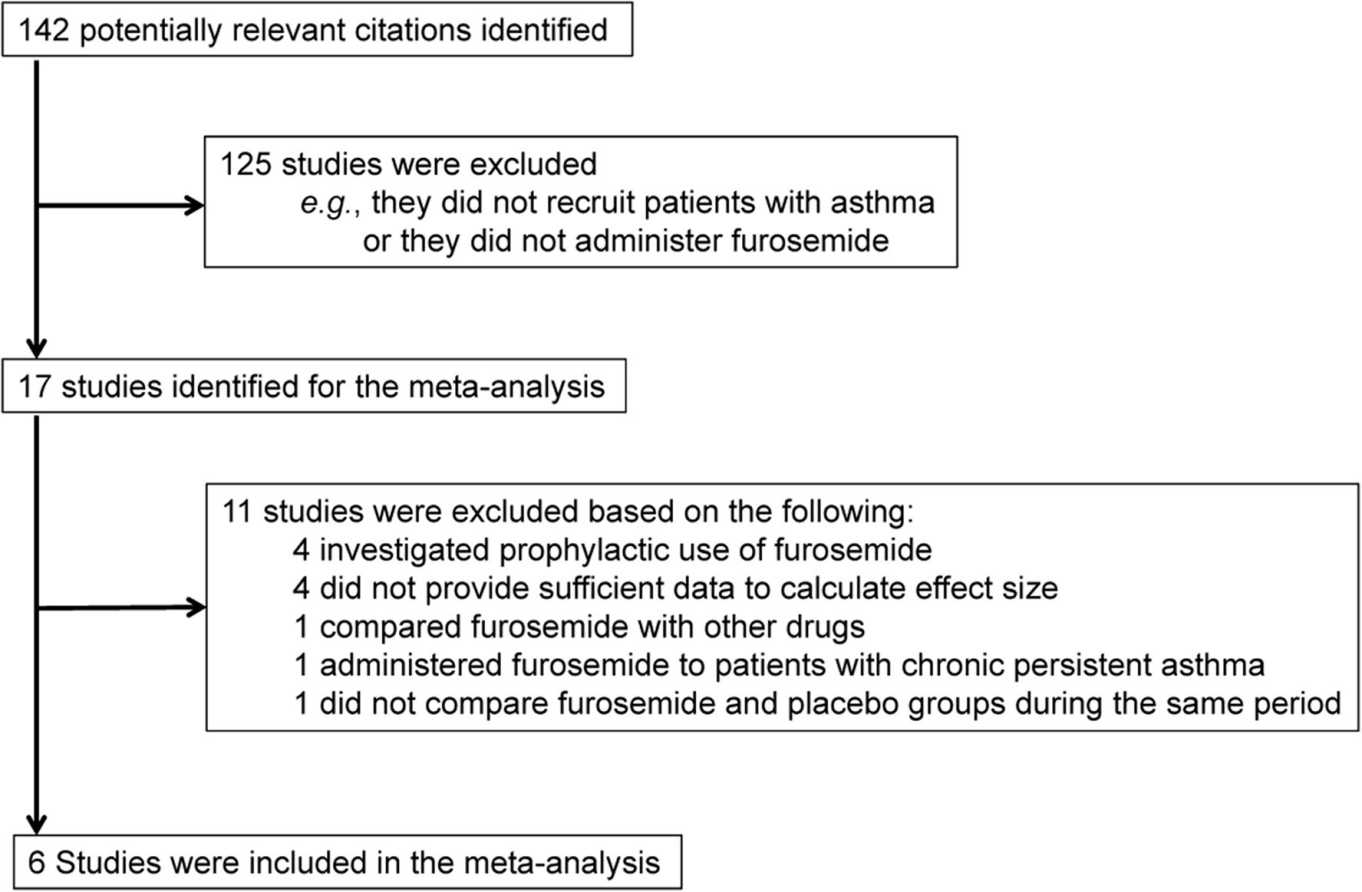
**Study selection procedure.**

We identified six studies using double-blinded, randomized control trial designs that evaluated inhaled furosemide in conjunction with standard treatments in patients experiencing asthma attacks [1-6] (Figure 1); a total of 78 patients received inhaled furosemide and 79 patients received a placebo (Tables 1 and 2). The mean age of patients ranged from 8.4 to 47 years [3,6]. In two studies, patients were administered 40 mg inhaled furosemide [1,2]; in one study, patients were administered 20 mg inhaled furosemide [6]; and in the three studies that recruited children, patients were administered either 1.0 mg/kg [4,5] or 10 mg/m^2^ [3] inhaled furosemide.

**Table 1:** **Trial characteristics**

			**Furosemide group**		**Placebo group**									
**Study (year)**	**Number of randomized patients**	**Number of patients completing study**	**Mean patient age (years)**	**Number of patients (male)**	**Mean patient age (years)**	**Number of patients (male)**	**Smoking**	**COPD**	**β-agonist dose**	**Inhaled furosemide dose**	**Expiratory airflow assessment time (minutes)**	**Spirometry measurement used**	**Severity**	**Hydrocortisone (mg)**
Alshehri and colleagues, 2005 [5]	39	39	8.4	19 (11)	8.5	20 (9)	N/A^a^	N/A^a^	0.15 mg/kg	1.0 mg/kg	30	PEFR, FEV1.0	Moderate	N/A
González-Sánchez and colleagues, 2002 [4]	20	20	9.8	10 (7)	10	10 (5)	N/A^a^	N/A^a^	0.15 mg/kg	1.0 mg/kg	30, 60	FEV1.0	Mild or moderate	N/A
Nannini and colleagues, 1992 [1]	20	16	31	7 (N/A)	41	9 (N/A)	N/A	N/A	2.5 mg	40 mg	15, 30	PEFR	N/A	N/A
Nuhoğlu and colleagues, 2006 [3]	32	32	8.6	16 (8)	8.4	16 (12)	N/A^a^	N/A^a^	0.15 mg/kg	10 mg/m^2^	N/A	PEFR	Mild or moderate	N/A
Ono and colleagues, 1997^b^ [6]	37	37	47	20 (7)	41	17 (8)	N/A	Exclude	N/A	20 mg	30, 60	PEFR, FEV1.0	Mild to severe	100
Pendino and colleagues, 1998^c^ [2]	42	42	38	6 (N/A)	34	8 (N/A)	Not >10 pack-years	Exclude	2.5 mg	40 mg	15, 30	PEFR	Mild or moderate	300

COPD, chronic obstructive pulmonary disease; FEV1.0, forced expiratory volume in 1 second; N/A, not available; PEFR, peak expiratory flow rate. ^a^Smoking and COPD histories were not available, although no smoking or COPD history was assumed because patients were children. ^b^Combination treatment in all trials was simultaneous administration of a beta-agonist plus furosemide, except for Ono and colleagues, in which patients in both groups received hydrocortisone succinate and aminophylline, followed 30 minutes later by either furosemide or placebo. ^c^Only subgroup data (pertaining to patients whose exacerbations lasted <8 hours) were available.

**Table 2:** **Trial results**

	**Furosemide**								**Placebo**							
	**PEFR**				**FEV1.0**				**PEFR**				**FEV 1.0**			
**Study (year)**	**Baseline airflow**	**SD**	**Post-treatment airflow**	**SD**	**Baseline airflow**	**SD**	**Post-treatment airflow**	**SD**	**Baseline airflow**	**SD**	**Post-treatment airflow**	**SD**	**Baseline airflow**	**SD**	**Post-treatment airflow**	**SD**
Alshehri and colleagues, 2005 [5]	59.0	22.0	84.9	14.0	58.5	14.5	80.2	13.9	57.2	25.4	80.7	17.4	56.7	17.3	77.8	19.1
Nuhoğlu and colleagues, 2006 [3]	178	65.9	222	66.1	N/A	N/A	183	51.7	218	60.3	N/A	N/A				
	**Baseline airflow**	**SD**	**Net airflow improvement above baseline**	**SD**	**Baseline airflow**	**SD**	**Net airflow improvement above baseline**	**SD**	**Baseline airflow**	**SD**	**Net airflow improvement above baseline**	**SD**	**Baseline airflow**	**SD**	**Net airflow improvement above baseline**	**SD**
González-Sánchez and colleagues, 2002 [4]	N/A	N/A	0.820	0.460	0.910	0.067	N/A	N/A	0.850	0.340	0.980	0.078				
Nannini and colleagues, 1992 [1]	147	68.0	269	89.7	N/A	N/A	234	82.0	316	56.2	N/A	N/A				
Ono and colleagues, 1997 [6]	171	20.0	205	45.1	1.18	0.13		178	25.0	198	21.3	1.32	0.74	N/A		
Pendino and colleagues, 1998 [2]	200	71.0	426	98.0	N/A	N/A	209	68.0	337	73.2	N/A	N/A				

FEV1.0, forced expiratory volume in 1 second; N/A, not available; PEFR, peak expiratory flow rate; SD, standard deviation.

**Figure 2:**
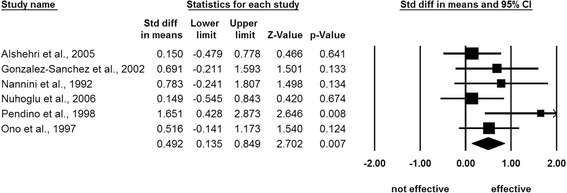
**Meta-analysis of randomized clinical trial studies.** A random effects model demonstrated significant effectiveness of inhaled furosemide for asthma attacks [1-6]. CI, confidence interval; Std, standard.

**Figure 3:**
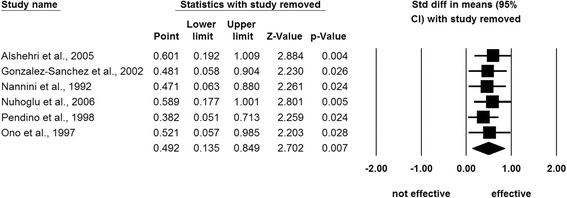
**Jackknife sensitivity analysis, excluding one study at a time.** All sensitivity analyses preserved the significant effectiveness of inhaled furosemide for asthma attacks [1-6]. CI, confidence interval; Std, standard.

Integrating the standardized mean difference in each study, a random effects model showed that inhaled furosemide had a significant positive effect on asthma attacks (*Z* = 2.70; 95% confidence interval, 0.14 to 0.85; *P* = 0.007) with a negligible heterogeneity (*I*^2^ = 16.82) (Figure 2 and Table 3). Subanalyses of the studies reporting the peak expiratory flow rate (*Z* = 2.23; *P* = 0.026; *n* = 68/70, inhaled furosemide/placebo) and the forced expiratory flow in 1 second (*Z* = 1.84; *P* = 0.066; *n* = 49/46, inhaled furosemide/placebo) values confirmed the significant effectiveness of inhaled furosemide for asthma attacks (Table 3). Jackknife sensitivity analyses confirmed the replicability of these findings (*P* <0.028) (Figure 3). No adverse events associated with furosemide inhalation were reported.

**Table 3:** **Meta-analyses of randomized controlled trials**

	**Number of studies**	**Furosemide group (** ***n*** **)**	**Placebo group (** ***n*** **)**	**Lower 95% CI**	**Upper 95% CI**	***Z*** **value**	***P*** **value**	***I*** ^***2***^
Whole studies	6	78	79	0.14	0.85	2.70	0.007	16.8
PEFR	5	68	70	0.058	0.90	2.23	0.026	30.4
FEV1.0	3	49	46	–0.027	0.83	1.84	0.066	8.16

CI, confidence interval; FEV1.0, forced expiratory volume in 1 second; PEFR, peak expiratory flow rate.

These results thus reveal a statistically significant improvement in airflow obstruction with no evident adverse events when inhaled furosemide was used as an adjunctive treatment for acute asthma exacerbation. The present study provides evidence supporting the addition of inhaled furosemide to conventional treatment in clinical situations.
